# Oral Morphine Use in South India: A Population-Based Study

**DOI:** 10.1200/JGO.2016.007872

**Published:** 2017-02-08

**Authors:** M.R. Rajagopal, Safiya Karim, Christopher M. Booth

**Affiliations:** **M.R. Rajagopal**, Trivandrum Institute of Palliative Sciences and Pallium India, Trivandrum, India; and **Safiya Karim** and **Christopher M. Booth**, Queen’s University, Kingston, Ontario, Canada.

## Abstract

**Purpose:**

Access to opioids for pain control is recognized as an urgent issue in low- and middle-income countries. Here we report temporal and regional trends in morphine use in Kerala, India.

**Methods:**

Oral morphine use data for the State of Kerala (2012 to 2015) was used to describe temporal trends, regional variation, and provider characteristics. Total morphine use was calculated for each district of Kerala to derive an annual per capita use rate (milligrams per capita). Each provider was classified as government, private, nongovernment organization (NGO), or NGO partnership.

**Results:**

Oral morphine use for Kerala was 1.32 mg/capita and increased over the study period 27% (from 1.23 mg/capita to 1.56 mg/capita). There was substantial variation in morphine use across districts (range, 0.49 mg/capita to 2.97 mg/capita; six-fold difference). This variation increased over time (19-fold difference in 2015). In 2015, 31% of morphine providers (51 of 167) were government institutions; they delivered 48% of total morphine in Kerala. Corresponding data for other providers are private institutions, 23% of centers and 13% of morphine; NGOs, 41% of centers and 34% of morphine; and NGO partnerships, 5% of centers and 4% of morphine. From 2012 to 2015, the total number of centers increased by 35%, from 124 to 167.

**Conclusion:**

Oral morphine use has increased over time in Kerala but remains substantially lower than estimated need. There is significant geographic variation of use. Efforts are needed to improve palliative care in Kerala and to reduce regional disparities in access to opioids.

## INTRODUCTION

Lack of access to palliative care is a global issue, with an urgent need for scale-up of services in low- and middle-income countries (LMICs).^1,2^ This is of increasing relevance in many LMICs, where cancer has emerged as a major threat to public health. Relief of cancer pain is considered by many organizations to be a basic human right, yet 66% of the world’s population has virtually no access to opioids for pain, and only 8% of the global population has consumption levels that are considered adequate.^3^ In 2009, high-income countries in North America, Europe, and Oceania, accounting for 18% of the global population, consumed 93% of the global morphine.^4^ Although pain relief is only one element of palliative care, global monitoring of opioid use is an objective and commonly used surrogate measure of access to palliative care.

More than 1 million new cases of cancer are diagnosed annually in India, which has a population of 1.2 billion.^5^ Because most patients in India are diagnosed with advanced disease, the cancer mortality rate is high, at 68% of the annual incidence.^6^ Estimates suggest that up to 80% of patients with advanced cancer will develop significant pain.^7^ Palliative care and health services research were highlighted as urgent research priorities in recent seminal overviews of cancer care in LMICs globally^2^ and specifically in India.^8^

A number of reports have described access to opioids in India at a national level.^1,3,9^ Several studies have also explored barriers to opioid use in India.^10-12^ However, to our knowledge, there are no published studies that have described details of opioid use and delivery at the state or regional level. Although country-level opioid use statistics are helpful for national policy, promotion of system change requires access to granular data for clinicians, educators, and policymakers on the ground. To address these important gaps in the literature, we undertook a population-based study in the southern Indian state of Kerala to describe temporal and regional trends in oral morphine use across districts and describe organizational delivery of oral morphine in Kerala.

## METHODS

### Study Setting

Kerala is located on the southwest coast of India and has a population of approximately 33 million. Kerala has the highest literacy rate (94% *v* 73% national rate), greatest life expectancy (74 years *v* 64 years), and lowest infant mortality rate in India.^13^ Kerala is widely recognized as a global leader in palliative care.^2^ Pivotal events in the development of palliative care in Kerala include the establishment of one of India’s first pain clinics at the Regional Cancer Centre of Trivandrum in 1986. A subsequent significant development occurred in 1993 with the establishment of the Pain and Palliative Care Society (PPCS) in Calicut, a charitable nongovernmental organization (NGO). PPCS established palliative care clinics, educational initiatives, and a community-based system of delivering home palliative care by a network of volunteers and health professionals. PPCS was declared a demonstration project by the World Health Organization in 1995. Pallium India (based in Kerala’s capital, Trivandrum) was created as a charitable trust in 2003 as a national advocacy and educational organization. The State Government of Kerala adopted a Palliative Care Policy in 2008, declaring palliative care as an integral component of standard health care. By that time Kerala was already leading India, with 83 of India’s 139 palliative care service providers.^12^ Additional international recognition for palliative care efforts in Kerala culminated in 2010 and 2012, when the Institute of Palliative Medicine at Calicut and the Trivandrum Institute of Palliative Sciences were recognized as WHO Collaborating Centres.

Palliative care in Kerala is currently delivered in a number of diverse settings by a variety of organizations. Government hospitals, private hospitals, and community-based NGOs deliver palliative care services to inpatients, to outpatients, and through mobile units at home. Oral morphine is the only opioid available in Kerala and can only be dispensed by an approved provider affiliated with a Recognized Medical Institution (RMI). Physicians may prescribe oral morphine only after having completed 10 days of hands-on training in pain relief and palliative care.^14^ All oral morphine dispensed in the state of Kerala is procured from licensed manufacturers, who in turn get raw morphine powder from the Government Opium and Alkaloid Works, the only source of legal medical morphine in the country. On an annual basis, each RMI must submit the morphine consumption statistics for the previous year along with an estimate for the subsequent year to the Office of the Drugs Controller. The Drugs Controller of Kerala is supported by an advisory panel of four palliative care physicians, including a coordinator (currently, the first author of this article) and a physician from each of the three geographic zones of the state.

### Data Sources

Morphine-use data reported in this study come from data submitted by each RMI to the Office of the Drug Controller for four consecutive years in 2012 to 2015. Total morphine use was calculated for each district in Kerala; this quantity was divided by the population of each district^13^ to derive an annual per capita use metric (milligrams of morphine per person). Each RMI was also classified as being operated by a government provider, private provider, or community-based NGO. RMIs that represented collaborative ventures between multiple organizations were further classified as NGO partnerships.

### Statistical Analysis

Comparison between groups was made using the χ^2^ test. Results were considered statistically significant at *P* value < .05. Analyses were performed using EXCEL and SAS version 9.3 (SAS Institute, Cary, NC).

## RESULTS

### Morphine Use by District and Over Time

**Fig 1:**
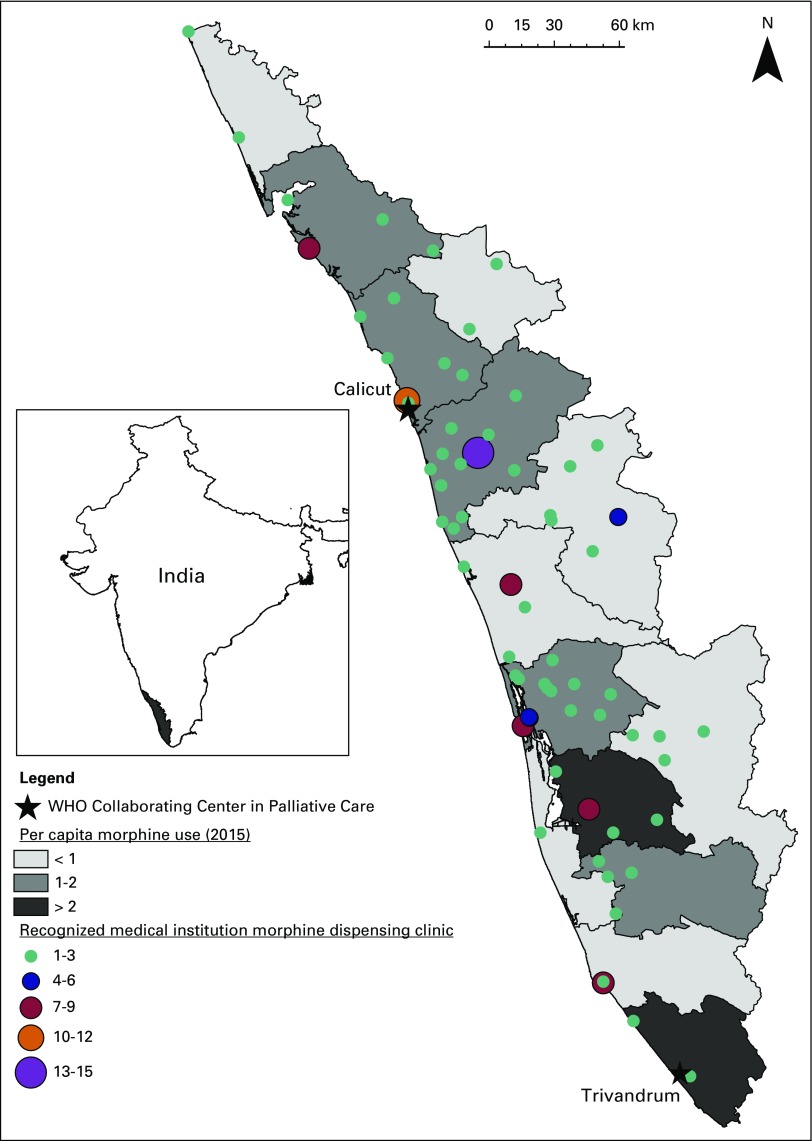
– Map of Kerala showing distribution of recognized medical institution morphine dispensing clinics and district-level per capita morphine-use rate in 2015. Clinic locations may reflect clusters of clinics in the same city.

During 2012 to 2015, annual per capita use for all of Kerala was 1.32 mg/person. During the study period, Kerala morphine consumption increased by 27% (from 1.23 mg/capita in 2012 to 1.56 mg/capita in 2015). As shown in Table 1, during 2012 to 2015 there was substantial variation in morphine use across the districts of Kerala, with a range from 0.49 mg/capita to 2.97 mg/capita (six-fold difference). Temporal data suggest that this district-level variation is increasing over time, with an observed eight-fold difference in 2012 and 19-fold difference in 2015. Geographic distribution of morphine-prescribing facilities and district-level per capita use of morphine are shown in Figure 1.

**Table 1:**
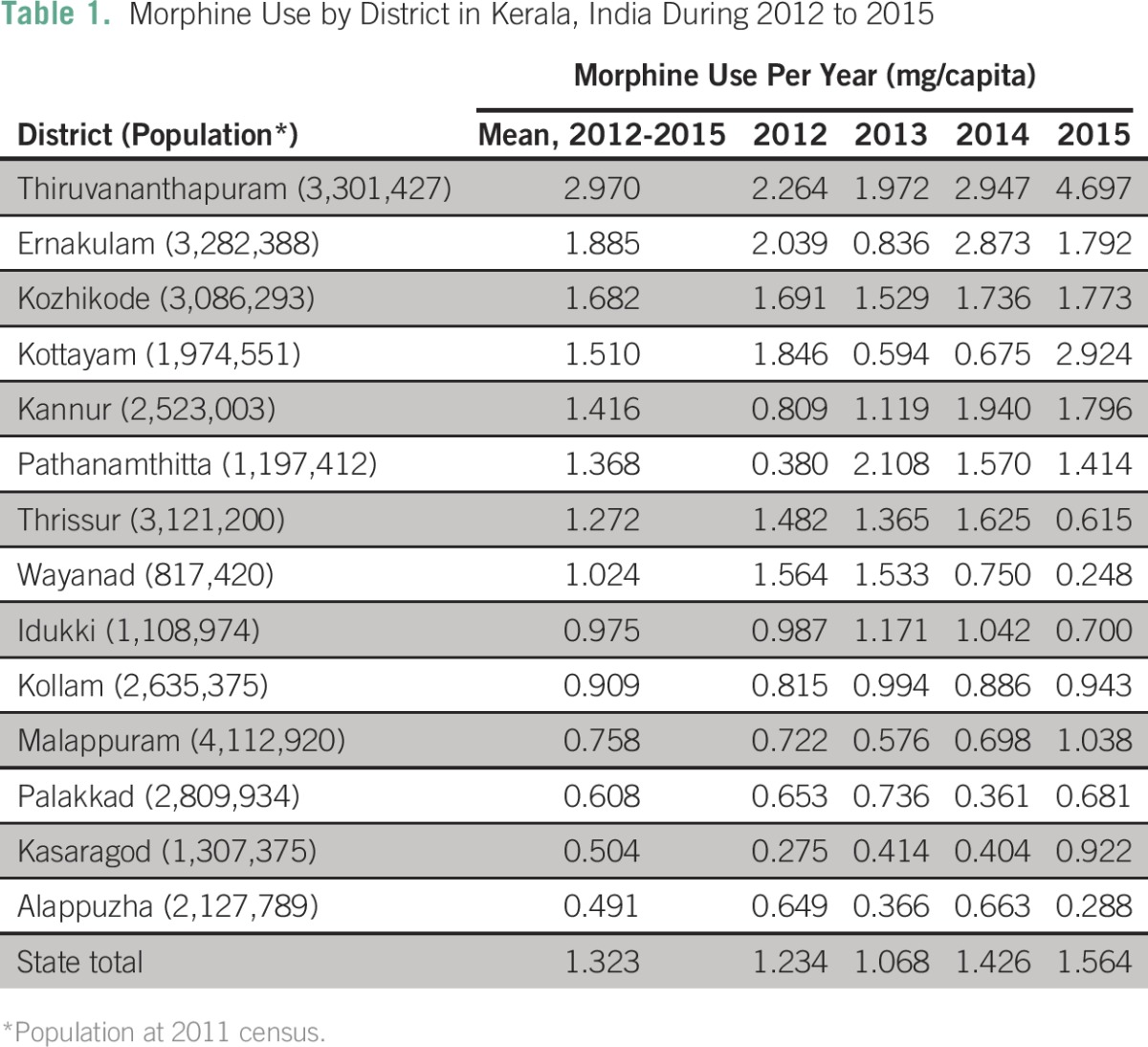
– Morphine Use by District in Kerala, India During 2012 to 2015

Table 2 indicates the substantial geographic variation in the number of morphine prescribing centers across districts (range, 2 to 34). The data in Table 2 demonstrate that there is no clear relationship between the number of prescribing centers and per capita morphine use.

**Table 2:**
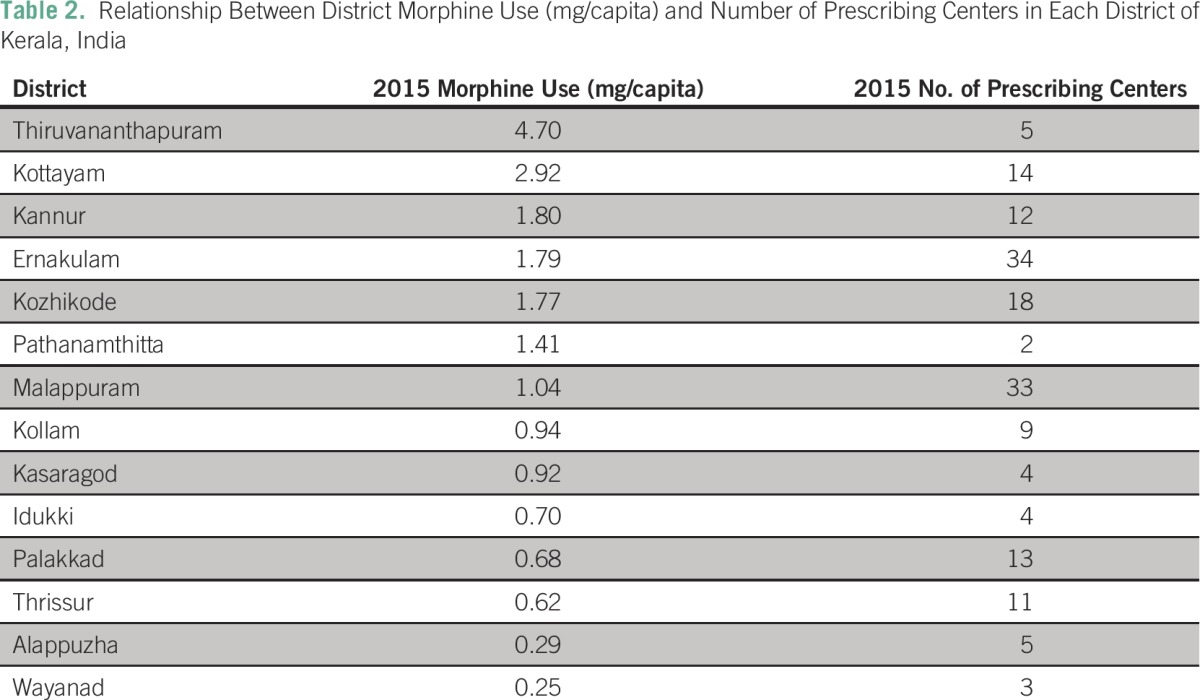
– Relationship Between District Morphine Use (mg/capita) and Number of Prescribing Centers in Each District of Kerala, India

### Characteristics of Morphine Providers in Kerala

During 2012 to 2015, the total number of centers in Kerala has increased by 35%. In 2015, there were 167 institutional providers of oral morphine in Kerala. Among these providers, 31% (51 of 167) were government institutions, 23% (39 of 167) were private institutions, 41% (68 of 167) were NGOs, and 5% (nine of 167) were NGO partnerships with government or private institutions. Although government institutions accounted for 31% of the palliative care centers in 2015, they delivered 48% of total morphine in the state. Corresponding data for other providers are: private institutions, 23% of centers and 13% of morphine; NGOs, 41% of centers and 34% of morphine; and NGO partnerships, 5% of centers and 4% of morphine.

**Fig 2:**
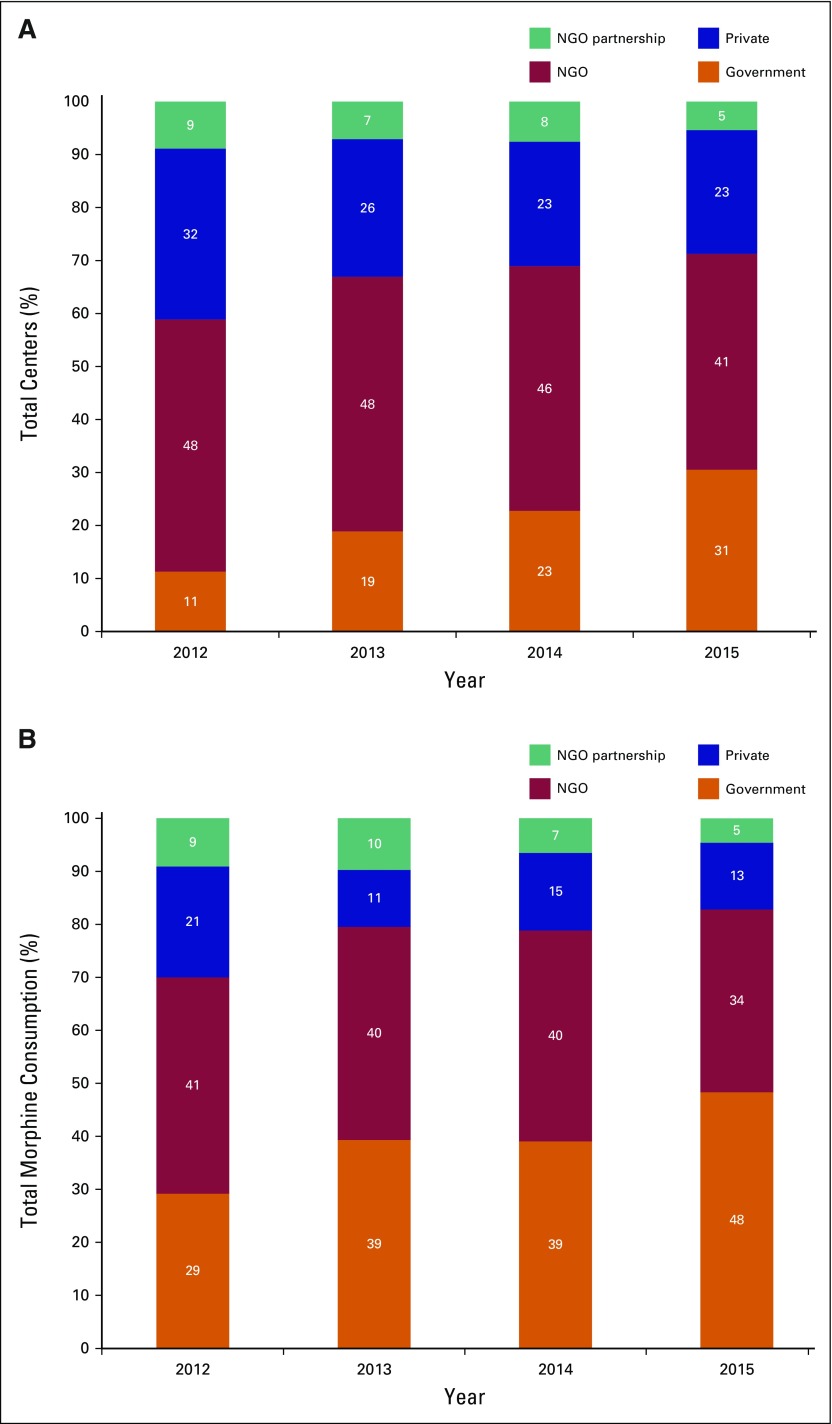
– Temporal trends in number of (A) palliative care centers and (B) volume of morphine prescribed in Kerala, India, 2012 to 2015 by provider organization. NGO, nongovernment organization.

Temporal trends in provision of palliative care services in Kerala are shown in Figure 2. The most notable trend noted between 2012 and 2015 is the substantial proportional increase in the number of government institutions (from 11% to 31% of total centers, *P* < .001) and volume of morphine prescribed by government institutions (from 29% to 48% of total morphine, *P* < .001).

## DISCUSSION

In this population-based study, we describe morphine use in the Indian state of Kerala during 2012 to 2015. Several important findings have emerged. First, during the study period, the mean per capita consumption of morphine has increased and was 1.56 mg in 2015. Second, within Kerala we have observed substantial variation in morphine use by district, and this regional variation seems to be increasing over time; there was a 19-fold difference in per capita use across districts in 2015. Third, this study demonstrates that morphine is delivered by a network of providers, including government, private sector, and NGOs. Fourth, although the number of centers and total morphine use in the government sector has increased over the study period, in 2015 one-third of morphine use for the state of Kerala was delivered by NGOs. Finally, although these data suggest that morphine use is higher in Kerala than elsewhere in India and that access within Kerala is improving over time, it is still substantially lower than the global mean consumption level (6.27 mg/capita) and the estimated needs of the Indian population.^15^

A number of studies have reported opioid consumption at the country level. Global mean morphine consumption in 2013 was 6.27 mg/capita; per capita consumption in India was 0.11 mg, ranking 113 of 139 countries.^15^ To put these figures in context, it is worth considering morphine equivalent (ME) rates of the highest-use countries, such as Canada (723 mg/capita ME), United States (718 mg/capita ME), Australia (454 mg/capita ME), United Kingdom (241 mg/capita ME), France (213 mg/capita ME), and Italy (204 mg/capita ME).^15^ A less commonly reported metric of opioid access is morphine equivalent use per cancer death. GLOBOCAN data suggest there were 683,000 cancer deaths in India in 2012.^16^ On the basis of the relative population of Kerala, there would be approximately 19,482 deaths in 2012; this translates to approximately 2,041 mg/cancer death. Comparable ME data from other countries include: Canada, 284,653 mg/cancer death; United States, 266,752 mg/cancer death; United Kingdom, 103,240 mg/cancer death; France, 57,651 mg/cancer death; and Italy, 24,287 mg/cancer death.

There is no universal agreement on the optimal use rate of oral opioids.^3^ Although the high rates in Canada and the United States reflect systems in which opioid overuse is well recognized, the rates in England, France, and Italy may serve as reasonable benchmarks for other jurisdictions. Our data demonstrate that although Kerala is often hailed as the leading light of palliative care in India, a tremendous shortfall in access to adequate pain control still exists. It is notable that our finding of 1.56 mg/capita of morphine used in Kerala during 2015 does not include intravenous morphine, although we believe it unlikely that our overall findings would be substantially altered with this added information.

Barriers to opioid accessibility in India and other LMICs are known to be complex and multifactorial. In a recent overview of opioid access, Cleary et al^10^ elegantly describe regulatory barriers in India that impede access to morphine, including: requirements for physicians to receive special authority/license to prescribe opioids, requirements for duplicate prescriptions/special prescription forms, prescription limits of 30 days, inability to prescribe opioids in an emergency situation by fax or telephone, and pharmacists not having the authority to correct a prescription with a technical error. Compounding these structural barriers is the fact that even when opioids are included on hospital/clinic formulary, they are often not available. LeBaron et al^11^ performed an in-depth study of barriers to cancer pain management at a large cancer hospital in South India. They found that, although morphine was more available at the study hospital than many other sites in India, access was limited to those patients seen by the palliative care service and that there were significant gaps in supply. The authors identified several key barriers, including: limited involvement of nurses in evaluating pain, lack of basic knowledge in pain control (ie, incorrectly identifying antiemetics or sedative medications as analgesics), misperceptions among staff that cancer pain was viewed as inevitable and largely unmanageable, and structural barriers (ie, patients were only given 1-month supply at a time, and family members would need to return to the clinic from far distances to obtain refills). In another overview of global barriers, Berterame et al^9^ describe absence of training/awareness in medical professionals, fear of dependence, restricted financial resources, issues in sourcing, cultural attitudes, fear of diversion, international trade controls, and onerous regulation as significant impediments to opioid accessibility. In their global overview, Cleary et al^17^ describe the cornerstone trinity that is needed to improve opioid accessibility in LMICs: medication availability, education, and policy reform.

Although India has shown substantial progress in recent years, it is estimated that only < 1% of the population currently has access to palliative care services.^18^ Important initiatives in India are currently needed to implement recent changes to the National Drugs and Psychotropic Substances Act by state governments. In the absence of a unified approach, NGOs, which are already struggling with limited resources, will have to take on the onerous task of obtaining funds and personnel for catalyzing government action across 29 states and six union territories. From an educational perspective, the Medical Council of India and the Indian Nursing Council must incorporate palliative care into undergraduate curricula. Finally, although the National Program in Palliative Care was created in 2012, because of a lack of budget allocation only a tiny part of the program has been implemented.

Kerala’s access to morphine, so much ahead of the rest of the country, has its roots in the development of the palliative care movement in the state. The movement, started as a nongovernment activity, came to be widely believed to be a social need and social responsibility, with numerous community-based initiatives being active in the field. In response to unmet educational needs, NGOs within Kerala developed several palliative care courses, which have been attended by hundreds of physicians and nurses from Kerala and elsewhere in India.

Current regulations in Kerala stipulate that any institution wishing to dispense oral morphine must have at least one physician with a minimum period of 10 days training in palliative care. This clause was established on the recommendation of palliative care pioneers in the state. Because oral morphine was an unknown entity for medical professionals, they reasoned that such training was necessary to avoid irresponsible prescribing. In retrospect, although this clause may have limited access to oral morphine, it may have protected against some of the opioid overuse problems that are now common in many high-income countries. This requirement for additional training in palliative care will likely continue until pain assessment and management are broadly incorporated into undergraduate medical and nursing curricula. Other elements that are essential to improve access to pain relief in Kerala include better integration of palliative care into routine health care and improved cooperation between government institutions and NGOs, so that deficiencies of one (ie, availability of trained doctors) can be augmented by the other.

To our knowledge, this is the first study to describe population-based temporal trends in morphine use across districts in an LMIC. Although the study results provide important insights, they must be considered in light of methodologic limitations. The morphine-use data represent morphine dispensed by providers to patients by year and not the actual volume consumed by patients in routine practice. It is also worth noting that these data do not include intravenous opioids, which are delivered in hospital settings. The study data do not include other oral opioids belonging to step 3 of the WHO ladder, because none are available for use in India. Although transdermal fentanyl is available India, its use in Kerala is exceedingly uncommon. District-level morphine data are based on provider address, not patient residence. Because many patients travel from throughout Kerala to attend the Regional Cancer Centre in Trivandrum, it is therefore likely that the rate of morphine use reported in Trivandrum district is artificially inflated by including patients from other districts. Finally, some morphine providers cannot easily be classified into one category of NGO, government, or private institution. For example, Trivandrum Institute of Palliative Sciences (which is classified as an NGO in the present analysis) provides services to numerous clinics located in the community as well as within various government hospitals. Despite these study limitations, the temporal trends and overall use rates offer important insights into the delivery of care in LMICs.

In summary, this population-based study describes temporal and regional trends in morphine use in Kerala. This study addresses a known gap in research that has been highlighted in recent prominent calls for improved palliative care in LMICs.^2,8^ Although morphine use is increasing in Kerala over time, there is a widening gap in access across districts that warrants attention. Although many positive steps have been made to improve palliative care in Kerala, these data demonstrate a substantial shortfall between current use and estimated need. Data from this study will provide a framework for ongoing monitoring and quality improvement initiatives in Kerala and elsewhere in India. The study also demonstrates how other jurisdictions in LMICs might use health administrative data to close the gap between evidence and practice.
